# Determining sensitivity and specificity of risk scores for QTc interval prolongation in hemato-oncology patients prescribed systemic antifungal therapy: a retrospective cross-sectional study

**DOI:** 10.1007/s11096-024-01788-w

**Published:** 2024-08-14

**Authors:** Julian Steinbrech, Till Klein, Stephanie Kirschke, Hanna Mannell, Sebastian Clauß, Thilo Bertsche, Dorothea Strobach

**Affiliations:** 1 Hospital Pharmacy, LMU University Hospital, Marchioninistr. 15, 81377 Munich, Germany; 2 Doctoral Program Clinical Pharmacy, LMU University Hospital, Marchioninistr. 15, 81377 Munich, Germany; 3 Department of Physiology, Institute for Theoretical Medicine, Faculty of Medicine, University of Augsburg, 86159 Augsburg, Germany; 4 Department of Cardiology, LMU University Hospital, Marchioninistr. 15, 81377 Munich, Germany; 5 DZHK (German Center for Cardiovascular Research), Partner Site Munich Heart Alliance, Munich, Germany; 6 Institute of Surgical Research at the Walter-Brendel-Center of Experimental Medicine, LMU University Hospital, Marchioninistr. 27, 81377 Munich, Germany; 7 Member of the European Reference Network for Rare, Low Prevalance and Complex Diseases of the Heart (ERN GUARD-Heart), Munich, Germany; 8 Interfaculty Center for Endocrine and Cardiovascular Disease Network Modelling and Clinical Transfer (ICONLMU), LMU Munich, Munich, Germany; 9 Department of Clinical Pharmacy, Leipzig University, Brüderstr. 32, 04103 Leipzig, Germany; 10 Drug Safety Center, University Hospital of Leipzig, Leipzig University, Brüderstr. 32, 04103 Leipzig, Germany

**Keywords:** Antifungal agents, Hemato-oncology, Long QT syndrome, Pharmaceutical care, Risk assessment

## Abstract

**Background:**

QTc interval prolongation can result in potentially lethal arrhythmias. One risk factor is QTc-prolonging drugs, including some antifungals often used in hemato-oncology patients. Screening tools for patients at risk have not yet been investigated in this patient population.

**Aim:**

Our aim was to evaluate the sensitivity and specificity of five QTc risk scores in hemato-oncology patients receiving systemic antifungal therapy.

**Method:**

Data were retrieved from an internal study database including adult hemato-oncology patients prescribed systemic antifungal therapy. Data on QTc-prolonging medication, risk factors for QTc prolongation, and electrocardiograms (ECG) were collected retrospectively for a period of 12 months. The QTc risk scores according to Tisdale, Vandael, Berger, Bindraban, and Aboujaoude as well as their sensitivity and specificity were calculated.

**Results:**

During the evaluated period, 77 patients were prescribed systemic antifungals resulting in 187 therapy episodes. Regarding therapy episodes, median age was 56 years (IQR 44–68), 41% (77) were female, and a median of 3 QTc-prolonging drugs were prescribed (range 0–6). ECGs were available for 45 (24%) of the therapy episodes 3–11 days after initiation of the antifungal therapy, 22 of which showed QTc prolongation. Regarding these 45 therapy episodes, sensitivity and specificity of the risk scores were calculated as follows: Tisdale 86%/22%, Vandael 91%/35%, Berger 32%/83%, Bindraban 50%/78%, Aboujaoude 14%/87%.

**Conclusion:**

The QTc risk scores according to Tisdale and Vandael showed sufficient sensitivity for risk stratification in the studied patient population. In contrast, risk scores according to Berger, Bindraban, and Aboujaoude cannot be considered suitable due to poor sensitivity.

## Impact Statements

- Several risk scores to determine the patient-specific risk of QTc prolongation have been published. In this study, four out of five investigated risk scores were found not to be suitable for risk stratification of QTc prolongation in hemato-oncology patients receiving systemic antifungals due to poor sensitivity or poor usability. Risk scores should therefore be evaluated before they are routinely used in a new patient group.
- The Tisdale risk score showed sufficient sensitivity, specificity, and practicability in this patient population while being easy to calculate and data required for calculation being generally available. Routine use of the Tisdale score could, thus, lead to an increase in drug therapy safety in hemato-oncology patients receiving systemic antifungals.

## Introduction

Prolongation of the frequency-adjusted QTc interval in the electrocardiogram (ECG) is associated with an increased risk of arrhythmias, in particular Torsade de Pointes (TdP) [1, 2]. TdP, although often asymptomatic, is potentially life-threatening and can lead to sudden cardiac death, with a mortality rate of approximately 10% [3–5]. TdP can occur as a rare but potentially serious adverse effect of drugs [1, 2]. According to a study from the United States, 10% of the population are prescribed drugs that can prolong the QTc interval [6]. At hospital admission 50–90% of patients have been found to take QTc-prolonging drugs [7, 8]. Approximately 24–28% of all intensive care unit (ICU) patients and 37% of hospitalized elderly patients are affected by QTc prolongation [1, 8]. Nonetheless, the risk for drug-induced QTc prolongation is underestimated and considered to be given too little attention in clinical practice [2, 9, 10].

Aside from drugs, there are several known risk factors for QTc prolongation. These include advanced age and female gender, cardiac comorbidities (including myocardial infarction, heart failure, and bradycardia), electrolyte imbalances (especially hypokalemia), as well as impaired renal or hepatic function [2, 4, 10]. In manifest QTc prolongation and TdP, more than one risk factor or the intake of more than one QTc-prolonging drug is almost always present [11].

Hemato-oncology patients are often affected by several risk factors for QTc prolongation. Older patients, representing the majority of hemato-oncology patients, usually receive polymedication [12, 13], which may increase the risk of QTc prolongation by adding QTc-prolonging effects or drug interactions. Electrolyte imbalances are common in cancer patients [14]. In addition, various anticancer drugs themselves have QTc-prolonging properties [14, 15]. Due to immunosuppression following chemotherapy or stem cell transplantation, this patient population has a high risk of developing life-threatening invasive mycoses, which is why antifungal drugs are an irreplaceable prophylactic or therapeutic strategy [16, 17]. However, several antifungal substances, in particular azole antifungals, are known to cause QTc prolongation. For pharmacists, the question regularly arises as to when appropriate warnings must be given to the physicians when several QTc-prolonging drugs are co-prescribed, while at the same time avoiding over-alerting.

Since the genesis of QTc prolongation is multifactorial, patient-specific risk stratification may be useful [18]. Various risk scores for QTc prolongation have been developed in different patient cohorts [19–27]. However, none of these risk scores have been investigated so far in hemato-oncology patients receiving systemic antifungal therapy. Application of risk scores to stratify the risk of QTc prolongation as a decision-making aid has already shown significant benefits in several studies regarding reductions in the risk of QTc prolongation and prescriptions of QTc-prolonging drugs as well as better adherence to warnings generated based on risk scores [28–30].

### Aim

The aim of this study was to evaluate the sensitivity and specificity of five QTc risk scores in hemato-oncology patients receiving systemic antifungal therapy and to identify a suitable screening tool with regards to their applicability in this vulnerable group of patients.

### Ethics approval

Ethics approval was obtained from the Ethics Committee of the LMU hospital Munich (20–241).

## Method

### Study design

For this descriptive cross-sectional study, data from patients receiving systemic antifungal therapy between 04/2021–03/2022 at the LMU hospital Munich, Germany, were collected from an internal hemato-oncology study database. Inclusion criteria were age > 18 years, hospital stay for at least 24 h, and therapy or prophylaxis of invasive mycoses with systemic antifungal drugs in patients with a diagnosis of hematological or oncological malignancy.

### Risk scores for QTc prolongation

We performed a literature review for published risk scores for QTc prolongation. The risk scores according to Tisdale [27], Vandael [21], Berger [22], Bindraban [23], and Aboujaoude [20] were selected for evaluation in this study (Table 1). Selection criteria were the availability of the required parameters and the transferability of the scores to different patient populations.

**Table 1:** QTc risk scores selected for calculation

Score	Tisdale et al. [27]		Berger et al. [22]		Bindraban et al. [23]		Aboujaoude et al. [20]	
Parameters and calculation	Age ≥ 68 years	1	Age 51–75 years	1	Age > 70 years	1	Age ≥ 65 years	1
	Female sex	1	Age ≥ 76 years	2	Use of antiarrhythmics	1	Female sex	1
	Loop diuretic	1	Female gender	1	eGFR < 60 ml/min/1.73m^2^	2	(Ischemic) cardiomyopathy and/or hypertension	2
	Serum potassium ≤ 3.5 mmol/l	2	Cardiac comorbidities Hypertension	2 2	Use of loop diuretics	3	Arrhythmia	2
	Admission QTc ≥ 450 ms	2	Diabetes mellitus	1	Serum calcium ≤ 2.14 mmol/l	3	Thyroid disturbances	1
	Acute myocardial infarction	2	eGFR ≤ 50 ml/min/1.73m^2^	1	Serum potassium 3.0–3.4 mmol/l	3	Potassium ≤ 3.5 mmol/l	1
	1 QTc prolonging drug	3	Potassium ≤ 2.5 mmol/l	2	Maximal past QTc 481–500 ms (within 1 year)	3	Calcium < 2.15 mmol/l	1
	≥ 2 QTc prolonging drugs	3	Potassium 2.6–3.5 mmol/l	1	Maximal past QTc > 500 ms (within 1 year)	7	Each known risk QT-drug (CredibleMeds®)	1
	Sepsis	3	Loop diuretics	2				
	Heart failure	3	Known risk QT-drugs (CredibleMeds®)	1	Serum potassium ≤ 2.9 mmol/l	7	Each possible risk QT-drug (CredibleMeds®)	0.5
Cut-off values	Low-Risk: Score < 7 Medium-Risk: Score 7–10 High-Risk: Sre ≥ 11		Low-Risk: Score < 6 High-Risk: Score ≥ 6		Low-Risk: Score < 5 High-Risk: Score ≥ 5		Low-Risk: Score < 5 High-Risk: Score ≥ 5	

Score	Vandael et al. [21]							
Parameters and calculation	Predicted probability π to have a QTc ≥ 450(♂)/470(♀) (X) ${\uppi }\left( {\text{X}} \right) = { }\frac{{{\text{e}}^{\mu } }}{{1 + {\text{e}}^{\mu } }}$ $$\begin{aligned} \mu = & \left( { - 0.192{\text{*x}}_{1} } \right) + \left( {2.096{\text{*x}}_{2} } \right) + \left( {2.368{\text{*x}}_{3} } \right) + \left( {0.682{\text{*x}}_{4} } \right) + \left( {0.091{\text{*x}}_{5} } \right) \\ & \; + \left( {0.053{\text{*x}}_{6} } \right) + \left( {0.332{\text{*x}}_{7} } \right) + \left( {0.553{\text{*x}}_{8} } \right) + \left( {0.066{\text{*x}}_{9} } \right) + \left( {0.509{\text{*x}}_{10} } \right) \\ & \; + \left( { - 0.565{\text{*x}}_{11} } \right) + \left( {0.419{\text{*x}}_{12} } \right) + \left( {0.480{\text{*x}}_{13} } \right) + \left( {0.124{\text{*x}}_{14} } \right) + \left( {0.298{\text{*x}}_{15} } \right) \\ & \; + \left( {0.802{\text{*x}}_{16} } \right) + \left( {1.121{\text{*x}}_{17} } \right) + \left( {0.141{\text{*x}}_{18} } \right) + \left( {0.088{\text{*x}}_{19} } \right) + \left( {0.064{\text{*x}}_{20} } \right) - 3.970 \\ \end{aligned}$$ x_1_ = availability of a previous ECG (within 1 year), x_2_ = QTc on a previous ECG ≥ 450 ms (♂)/470 ms (♀), x_3_ = QTc on a previous ECG ≥ 500 ms, x_4_ = age ≥ 65 years, x_5_ = availability of BMI, x_6_ = BMI ≥ 30 kg/m^2^, x_7_ = availability of potassium measurement, x_8_ = potassium ≤ 3.5 mmol/l, x_9_ = availability of calcium measurement, x_10_ = calcium < 2.15 mmol/l, x_11_ = availability of eGFR measurement, x_12_ = eGFR ≤ 30 ml/min/1.73m^2^, x_13_ = availability of CRP measurement, x_14_ = CRP > 5 mg/l, x_15_ = history of (ischemic) cardiomyopathy and/or hypertension, x_16_ = history of arrhythmia, x_17_ = liver failure, x_18_ = neurological disorders, x_19_ = the number of drugs in list 1 of CredibleMeds®, x_20_ = the number of drugs in list 3 of CredibleMeds®							
Cut-off values	Low-Risk: π(X) < 0.035 High-Risk: π(X) ≥ 0.035							

*BMI* Body mass index, *CRP* c-reactive protein, *eGFR* estimated glomerular filtration rate, *ECG* electrocardiogram

### Data collection and score calculation

Additional data were collected retrospectively and included clinical, laboratory, and medication data. Clinical and laboratory data were obtained from the electronic patient information system (SAP i.s.h.med, Cerner Corporation, North Kansas City, USA). Data on patient medication were retrieved from the electronic prescribing software Meona (Mesalvo GmbH Freiburg, Germany) or, for periods before its implementation, from the scanned paper chart in SAP.

The complete medication and the respective classification regarding risk of QTc prolongation according to CredibleMeds® [15], as well as patient demographic data, diagnoses, and laboratory parameters relevant for the calculation of each of the risk scores were recorded for the day of initiation of the antifungal drug. The five risk scores were also calculated retrospectively for this day.

ECG data were obtained in the period from one year before the start of systemic antifungal therapy to the end of the inpatient stay. This period was chosen as the QTc interval of the last ECG within one year before the score calculation is required for the Vandael risk score [21]. The QTc intervals (Bazett [31]) were taken from the ECG reports generated by the ECG devices used at the hospital.

QTc prolongation was defined as a QTc interval of ≥ 450 ms in men and ≥ 470 ms in women or an increase of ≥ 30 ms to the QTc interval of a previous ECG (maximum 3 days before the start of antifungal therapy). QTc prolongation during antifungal therapy was defined as such if it occurred within 3–11 days of antifungal drug initiation (ECGs recorded during this period are hereinafter referred to as ECG_3–11_). This time-frame was chosen in analogy to the validation of the RISQ-PATH score by Vandael et al. [19].

In case of missing data, a normal value or non-existence of the parameter was assumed. This represents the common method used in the validation of QTc risk scores [22, 32]. If patients were admitted more than once during the analysed period or started on a different antifungal drug, each therapy episode was recorded individually.

### Statistics

Descriptive statistics were performed using Microsoft Excel 2016 (Seattle, WA, USA). Quantitative variables were reported as median and interquartile range (IQR) or range, and qualitative variables were reported as median and frequency distribution. Significances for quantitative variables were calculated after testing for normal distribution using Kolmogorov–Smirnov tests with the unpaired two-sample t-test (all samples tested showed normal distribution). Significances for qualitative dichotomous variables were calculated using Fisher’s exact tests. The significance level was set at α = 0.05. Sensitivity [true positive/(true positive + false negative)] and specificity [true negative/(true negative + false positive)] as well as positive (PPV) [true positive/(true positive + false positive)] and negative (NPV) [true negative/(true negative + false negative)] predictive values of the risk scores were determined. Due to the study design as a retrospective data analysis of a given patient population, sample size calculation was not possible.

## Results

### Patient characteristics

During the 12-month analysis period, 77 individual patients were identified. Within this patient population, 187 therapy episodes with systemic antifungal drugs were initiated. Patient characteristics are shown in Table 2.

**Table 2:** Patient characteristics

	Individual patients (n = 77)	Therapy episodes (n = 187)
Age [years] (median (IQR))	59 (48–71)	56 (44–68)
≥ 68 years of age (n (%))	20 (26)	44 (24)
Female (n (%))	28 (36)	77 (41)
eGFR < 60 ml/min/1.73m^2^ (n (%))	*	36 (19)
Number of QTc-prolonging drugs (median (range))	*	3 (0–6)
**Underlying disease**		
Leukemia (n (%))	*	162 (87)
Lymphoma (n (%))	*	21 (11)
Other malignancies (n (%))	*	5 (3)

*IQR* interquartile range, *eGFR* estimated glomerular filtration rate

^*^Not applicable, as these parameters can change between several therapy episodes of a patient

Apart from antifungal drugs, the five most prescribed drugs associated with a risk of QTc prolongation were pantoprazole (83%, n = 156) (Conditional Risk of TdP), piperacillin/tazobactam (22%, n = 42) (Conditional Risk of TdP), granisetron (17%, n = 32) (Possible Risk of TdP), torasemide (13%, n = 24) (Conditional Risk of TdP), and metoclopramide (12%, n = 23) (Conditional Risk of TdP) [15].

### ECGs and systemic antifungals

ECGs recorded during the inpatient stay were available in 104 of the 187 therapy episodes (56%). In 45 of the 187 (24%) therapy episodes, an ECG_3–11_ was available. In 59 (32%) therapy episodes, ECGs were recorded before the defined period (42 (22%)) and/or > 11 days after antifungal therapy initiation (24 (13%)). In 22 of 45 (49%) cases with an ECG_3–11_, a QTc prolongation was present. No TdP arrhythmias were recorded. QTc prolongations and antifungal drugs used in these therapy episodes are shown in Table 3.

**Table 3:** Distribution of antifungal drugs in the studied therapy episodes and registered QTc prolongations

Antifungal drug and classification according to CredibleMeds® [15]	Number of therapy episodes	Number of ECGs	Number of therapy episodes with ECG_3–11_	Number of ECG_3–11_	Number of QTc prolongations
**Known Risk of TdP**	**3**	**3**	**0**	**0**	**0**
Fluconazole	3	3	0	0	0
**Conditional Risk of TdP**	**137**	**147**	**34**	**89**	**15**
Voriconazole	44	52	9	33	5
Posaconazole	62	50	15	27	5
Amphotericin B*	31	44	10	29	5
**No risk known**	**48**	**36**	**11**	**21**	**7**
Isavuconazole	10	3	0	0	0
Caspofungin*/Micafungin	38	33	11	21	7

*ECG*_3–11_ ECG recorded within 3–11 days of antifungal initiation

^*^One therapy episode consisted of a dual therapy with Caspofungin and Amphotericin B

In all three therapy episodes in which fluconazole was prescribed, an ECG was recorded within 24 h before, but not after therapy initiation. QTc prolongations were thus not detectable for any of the patients receiving fluconazole.

### Therapy episodes with ECG available

Table 4 presents the risk factors for QTc prolongation recorded for the ECG_3–11_ subgroups with and without QTc prolongation.

**Table 4:** Occurrence of risk factors for QTc prolongation within subgroups of therapy episodes with ECG_3–11_

	Therapy episodes with ECG without QTc prolongation (n = 23)	Therapy episodes with ECG with QTc prolongation (n = 22)	*p*
Age [years] (median (IQR))	49 (31–68)	65 (56–74)	0.066
Age ≥ 68 years (n (%))	4 (17)	8 (36)	0.098
Female (n (%))	14 (61)	5 (23)	0.009
BMI (median (IQR))	25.7 (19.7–31.7)	24.6 (21.2–28.0)	0.220
Renal insufficiency (eGFR < 60 ml/min/1.73 m^2^ (n (%))	3 (13)	5 (23)	0.216
Ischemic cardiomyopathy (n (%))	0 (0)	2 (9)	0.233
Hypertension (n (%))	4 (17)	7 (32)	0.208
Heart failure (n (%))	3 (13)	6 (27)	0.066
Arrhythmias (n (%))	2 (9)	5 (23)	0.105
Occurrence of myocardial infarction during current inpatient stay (n (%))	0 (0)	2 (9)	0.233
Diabetes mellitus (n (%))	4 (17)	3 (14)	0.252
Thyroid dysfunction (n (%))	11 (48)	9 (41)	0.095
Neurological diseases (n (%))	3 (13)	5 (23)	0.270
Occurrence of hepatic failure during current inpatient stay (n (%))	0 (0)	0 (0)	n.a
Occurrence of sepsis during current inpatient stay (n (%))	0 (0)	0 (0)	n.a
Hypokalemia (current serum potassium ≤ 3.5 mmol/l)* (n (%))	5 (22)	2 (9)	0.049
Hypocalcemia (current serum calcium < 2.15 mmol/l)* (n (%))	2 (9)	6 (27)	0.038
Current CRP > 5 mg/dl* (n (%))	7 (30)	7 (32)	0.113
QTc interval ≥ 450 ms within 3 days before the start of therapy (n (%))	2 (9)	4 (18)	0.186
QTc interval ≥ 450 ms (♂)/470 ms (♀) within 1 year before the start of therapy (n (%))	10 (44)	13 (59)	0.089
Number of QTc-prolonging drugs (median (range))	3 (2–6)	3 (1–5)	0.692

*BMI* Body mass index, *CRP* c-reactive protein, *eGFR* estimated glomerular filtration rate, IQR: interquartile range

^*^Up to a maximum of 48 h before the start of antifungal therapy

### Risk score calculation

**Fig. 1:**
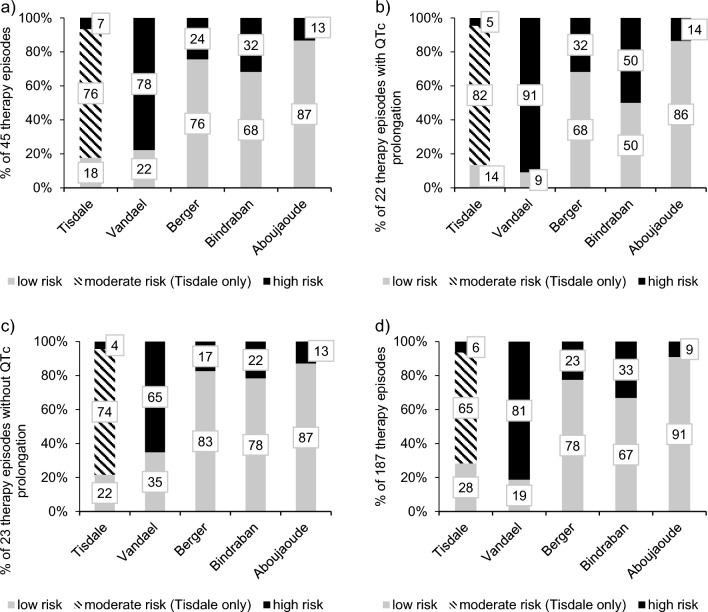
Distribution of risk score categories among antifungal therapy episodes:** a** 45 therapy episodes with ECG_3–11_; **b** 22 therapy episodes with ECG_3–11_ and QTc prolongation; **c** 23 therapy episodes with ECG_3–11_ and no QTc prolongation; **d** All 187 therapy episodes

For all 45 therapy episodes with an ECG_3–11_, the five selected risk scores (Table 1) were calculated. Results per risk category are shown in Fig. 1a. The Tisdale and Vandael risk scores each classified a large proportion of patients (37 (83%) and 35 (78%), respectively) as being at risk for QTc prolongation. In contrast, Berger, Bindraban, and Aboujaoude scores assessed 76% (34), 64% (29), and 87% (39) of patients, respectively, as being at low risk for QTc prolongation.

Figure 1b shows the distribution of score categories for the ECG_3–11_ subgroup with QTc prolongation (n = 22). The Tisdale and Vandael scores categorized 19 (86%) and 20 (91%) patients as being at high risk, respectively. In contrast, the Berger, Bindraban, and Aboujaoude scores categorized only 32% (7), 50% (11), and 14% (3), respectively, as high risk, resulting in calculated sensitivities of the risk scores of 86% (Tisdale), 91% (Vandael), 32% (Berger), 50% (Bindraban), and 14% (Aboujaoude), respectively.

The distribution of risk score categories in the 23 therapy episodes without QTc prolongation is shown in Fig. 1c. Tisdale and Vandael scores categorized 5 (22%) and 8 (35%) of the patients as low risk. Berger, Bindraban, and Aboujaoude scores categorized 83% (19), 78% (18), and 87% (20), respectively, as low risk, resulting in calculated specificities of the risk scores of 22% (Tisdale), 35% (Vandael), 83% (Berger), 78% (Bindraban), and 87% (Aboujaoude), respectively.

PPV of the risk scores were 51% (Tisdale), 57% (Vandael), 64% (Berger), 69% (Bindraban), and 50% (Aboujaoude), whereas NPV were 63% (Tisdale), 80% (Vandael), 56% (Berger), 62% (Bindraban), and 51% (Aboujaoude), respectively.

Figure 1d shows the distribution of score categories among all 187 therapy episodes. In comparison to the therapy episodes with an ECG_3–11_, similar results were obtained.

## Discussion

In this retrospective analysis of 187 antifungal therapy episodes in hemato-oncology patients, we evaluated for the first time the use of five QTc risk scores in this patient group with the aim of identifying patients with an increased risk for QTc prolongation and to minimize the risk for potentially life-threatening ventricular tachycardia through targeted interventions. QTc prolongations occurred in 22 of 45 therapy episodes (49%), where an ECG_3–11_ was recorded and were significantly associated with the presence of hypocalcemia. The scores according to Vandael and Tisdale achieved the best results regarding sensitivity with 91% and 86%, respectively.

### Performance of the investigated risk scores

In the studied patient population, the results of the risk scores calculated showed a pronounced heterogeneity. To some extent, this is to be expected, as the risk scores were developed and validated in different patient populations [20–23, 27]. Hence, the parameters generated in the original patient population which are included in the scores are probably not fully congruent with the risk factors relevant for hemato-oncology patients. This presumption was confirmed when comparing the risk factors of patients with and without QTc prolongation in an ECG_3–11_ (Table 4). Established risk factors such as hypokalemia were less frequent in therapy episodes in which QTc prolongation occurred than in those in which QTc prolongation did not occur.

As QTc prolongations are associated with a risk of potentially life-threatening arrhythmias, a high sensitivity of the risk scores is important. In this respect, the risk scores according to Vandael and Tisdale can be considered suitable for risk stratification. Regarding specificity, however, the scores only achieved moderate results leading to some patients being falsely classified as at risk for QTc prolongation. In practice, this could potentially result in additional interventions for patients who are not at risk. Interventions for patients deemed as at risk for QTc prolongation are usually limited to noninvasive methods, such as recording an ECG. In addition, Poncet et al. showed that increased ECG monitoring is an inexpensive measure leading to a reduced risk of TdP and TdP-related sudden cardiac death in psychiatric patients [33]. Therefore, authors of various risk scores conclude that high specificity can be dispensed with in favor of high sensitivity for detecting the risk of QTc prolongation [19, 21, 22, 32]. Due to the low sensitivity achieved, the risk scores according to Berger, Bindraban, and Aboujaoude cannot be considered suitable for risk stratification in this patient population.

While the risk score according to Vandael displayed the highest sensitivity, the Tisdale score has several advantages, such as its comparatively simple structure, the general availability of the parameters, and the possibility of a manual calculation. In comparison, the Vandael score is composed of a large number of parameters, of which some are often not available, and not manually calculable.

Overall, in our opinion, the Tisdale risk score is the most suitable for risk stratification of QTc prolongation in hemato-oncology patients prescribed systemic antifungal therapy.

### Antifungal therapy and QTc prolongation

Comparing the risk factors of patients with and without QTc prolongation, there were few statistically significant differences (Table 4). The data support the assumption that not one risk factor but the interaction of several factors is decisive for QTc prolongations. A limitation is the small number of patients in both groups. Hypocalcemia was the only risk factor significantly associated with QTc prolongation in this population. As hypocalcemia is only incorporated as a risk factor in the Vandael, Bindraban, and Aboujaoude risk scores, it might be interesting to investigate whether incorporating hypocalcemia into other risk scores would lead to an increase of sensitivity or specificity in hemato-oncology patients.

In this study, seven QTc prolongations occurred in patients receiving caspofungin or micafungin. This result is striking in that, according to CredibleMeds®, there is currently no evidence of a QTc-prolonging potential for these drugs [15]. However, due to retrospective data collection, it was not possible to determine whether the respective treatment decision for these drugs was potentially made due to a patient’s already increased risk of QTc prolongation or for another reason. It should be noted that the aim of this study was not to investigate, whether the systemic antifungals used in the population studied were themselves associated with a risk of QTc prolongation, as the presence of other risk factors for QTc prolongation, such as other QTc-prolonging medication, would interfere with the analysis. Furthermore, the potential of systemic antifungals to induce QTc prolongation and TdP is well documented in the literature, particularly for the azole antifungals (excluding isavuconazole, which is known to shorten the QTc interval) [15, 34–36].

### Importance of ECG monitoring

The European guideline for cardio-oncology recommends ECG monitoring at the start of and during therapy with QTc-prolonging drugs [37]. However, the recommended ECG checks are only performed in 39% of patients who are prescribed a drug with a high risk of QTc prolongation, as shown in a meta-analysis of 14 studies from different disciplines [38]. Due to the retrospective design of this study, it was not possible to actively recommend ECGs, nor could reasons for or against recording ECGs be determined. Since the decision to record an ECG may have been made in patients with an increased risk for QTc prolongation, it cannot be ruled out that this ECG-related selection bias may have influenced the analyses in this study.

Missing data, which could not be completed due to the retrospective design are a limitation of this study. For instance, the limited availability of ECGs should be noted. Another potential limitation is the automatic measurement of QTc intervals by the ECG devices. While some authors prefer manual measurement of the QTc interval due to a risk of misinterpretation with automatically calculated QTc intervals [39], other evaluations showed that non-cardiology physicians often do not calculate and interpret manually determined QTc intervals correctly [40, 41]. Since automatically calculated QTc intervals are widely used in everyday clinical practice [42], automatically calculated QTc intervals were used in this study.

The strength of this study is that it deals with real-life data over a period of one year, including all hemato-oncology patients receiving systemic antifungal drugs. The data thus reflect regular everyday clinical practice. After conducting a comprehensive literature search for risk scores for QTc prolongation we aimed to include a broad selection of scores in this study. Furthermore, documentation of a large number of risk factors for QTc prolongation was carried out in order to characterize the risk as precisely as possible.

Risk scores can serve to identify patients at risk for QTc prolongation in hemato-oncology patients receiving antifungal therapy. One option for integrating risk scores into clinical practice is to incorporate them into a clinical decision support system (CDSS). For the Tisdale risk score, this resulted in a reduced risk of QTc prolongation and fewer prescriptions of QTc-prolonging drugs on cardiology wards in a prospective study [28]. In another study, a reduction in the prescription of QTc-prolonging drugs and an increase in interventions to reduce the risk of QTc prolongation were demonstrated using a risk score-based CDSS [43]. Warnings based on risk scores when prescribing QTc-prolonging drugs resulted in better overall prescriber adherence compared with traditional CDSS and thus may counteract ignoring warnings and over-alerting [30]. Building on the results obtained in this study, integration of the Tisdale risk score into the hospital’s electronic medication record would be a promising way to increase drug therapy safety in hemato-oncology patients receiving systemic antifungal therapy. This approach should be further investigated in subsequent prospective studies. Furthermore, since hemato-oncology patients are exposed to a number of risk factors for QTc prolongations as well as other arrhythmia-inducing drugs (e.g., antiemetics, anti-cancer drugs, antibiotics [15, 44]), the use of QTc risk scores should be investigated in a wider population of hemato-oncology patients.

## Conclusion

The Tisdale risk score is a suitable risk score to stratify the risk of QTc prolongation in hemato-oncology patients prescribed systemic antifungal therapy due to a high sensitivity and good applicability in clinical practice. Integration of the risk score into practice could increase drug therapy safety by timely identification of patients at risk with the implementation of targeted ECG monitoring as well as a reduction of additional risk factors.
